# Amphetamine increases vascular permeability by modulating endothelial actin cytoskeleton and NO synthase via PAR-1 and VEGF-R

**DOI:** 10.1038/s41598-024-53470-w

**Published:** 2024-02-13

**Authors:** Julia Böttner, Tina Fischer-Schaepmann, Sarah Werner, Sarah Knauth, Heinz-Georg Jahnke, Holger Thiele, Petra Büttner

**Affiliations:** 1 Department of Cardiology, Heart Center Leipzig at Leipzig University, Strümpellstr. 39, 04289 Leipzig, Germany; 2 Institute for Orthodontics, Leipzig University, Liebigstr. 21, 04103 Leipzig, Germany; 3 Center for Biotechnology and Biomedicine at Leipzig University, Deutscher Platz 5, 04103 Leipzig, Germany

**Keywords:** Cell death, Cell signalling, Cytoskeleton

## Abstract

Abuse of amphetamine-type stimulants is linked to cardiovascular adverse effects like arrhythmias, accelerated atherosclerosis, acute coronary syndromes and sudden cardiac death. Excessive catecholamine release following amphetamine use causes vasoconstriction and vasospasms, over time leading to hypertension, endothelial dysfunction or even cardiotoxicity. However, immediate vascular pathomechanisms related to amphetamine exposure, especially endothelial function, remain incompletely understood and were analyzed in this study. Pharmaco-pathological effects of acute d-amphetamine-sulfate (DAM) were investigated ex vivo using contraction–force measurements of rat carotid artery rings and in vitro using label-free, real-time electrochemical impedance spectroscopy (EIS) on endothelial and smooth muscle cells. Specific receptor and target blocking was used to identify molecular targets and to characterize intracellular signaling. DAM induced vasodilation represented by 29.3±2.5% decrease in vascular tone (*p*<0.001) involving vascular endothelial growth factor receptor (VEGF-R) and protease activated receptor 1 (PAR-1). EIS revealed that DAM induces endothelial barrier disruption (−75.9±1.1% of initial cellular impedance, *p*<0.001) also involving VEGF-R and PAR-1. Further, in response to DAM, Rho-associated protein kinase (ROCK) mediated reversible contraction of actin cytoskeleton resulting in endothelial barrier disruption. Dephosphorylation of Serine1177 (−50.8±3.7%, *p*<0.001) and Threonine495 (−44.8±6.5%, *p*=0.0103) of the endothelial NO synthase (eNOS) were also observed. Blocking of VEGF-R and PAR-1 restored baseline eNOS Threonine495 phosphorylation. DAM induced vasodilation, enhanced vascular permeability and actin cytoskeleton contraction and induced eNOS hypophosphorylation involving VEGF-R, PAR-1 and ROCK. These results may contribute to a better understanding of severe adverse cardiovascular effects in amphetamine abuse.

## Introduction

Worldwide abuse of amphetamine-type stimulants like methamphetamine and its primary metabolite amphetamine is dramatically increasing independently of age, sex or ethnicity^1^. It is well known, that drug abuse in general affects the central nervous system causing structural and cognitive alterations^2–4^ but besides, has multiple effects on the cardiovascular system^5^. Frequent use of amphetamine-type stimulants can cause severe adverse cardiovascular events^6,7^ including Takotsubo syndrome, dilated cardiomyopathy, congestive heart failure and severe left ventricular dysfunction^8^, accelerated atherosclerosis, acute coronary syndromes and sudden cardiac death^1,2,5^. Amphetamine-based therapy of attention-deficit hyperactivity disorder has side effects like elevated heart rate and blood pressure^9^, vasospasms or Raynaud syndrome^10^. These mild to severe adverse cardiovascular events may result from iterative vascular damage due to amphetamine use^10^. Catecholamine release is considered to mediate these cardiovascular effects of amphetamine-type stimulants. At worst, this may cause an imbalance in oxygen supply and demand, limiting oxygen availability to the myocardium, eventually leading to myocardial hypertrophy, necrosis and fibrosis^11^. Further, high catecholamine levels cause vasoconstriction and vasospasms leading to hypertension and endothelial dysfunction resulting in acute coronary syndromes^5,11^. Next to these secondary effects of amphetamine, immediate direct effects on the vascular endothelium could cause sustainable damage to the vascular structure and function. Vascular permeability in pro-inflammatory signaling predominantly involves histamine receptor 1 (H1-R), protease activated receptor 1 (PAR-1) and vascular endothelial growth factor receptor (VEGFR) which regulate Ras homolog family member A/Rho-associated protein kinase (RhoA/ROCK) and thus cytoskeletal modulation and endothelial nitric oxide synthase (eNOS) and there with vasodilation. A strict regulation of these pathways ensures circulatory homeostasis and thereby proper cell and organ nourishment^12^. Dysfunctional vascular permeability, e.g. mediated by repeatedly induced hyperpermeability, can cause severe adverse events such as disturbed tissue homeostasis, and alterations in adhesion, platelet activation, vasotonus or ischemia–reperfusion injury^12–14^. Underlying molecular pathomechanisms of drug-induced vascular adverse events remain incompletely understood. Therefore, this study aimed to elucidate amphetamine specific signaling and alterations in ex vivo blood vessels and the main cells of the vascular system, endothelial and smooth muscle cells.

## Results

### DAM induced vasodilation in rat carotid rings

**Figure 1:**
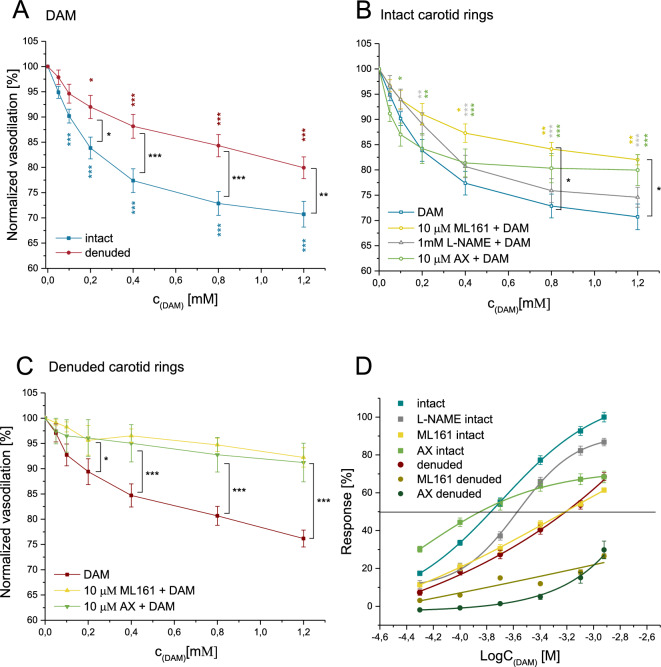
Vasodilation in response to DAM in intact and denuded rat carotid rings. DAM induced vasodilation is displayed as percentage of maximal contraction (induced by 300nM phenylephrine) in: (**A**) intact vs. endothelium-denuded carotid rings, (**B**) intact and (**C**) denuded carotid rings pre-treated with 10µM ML161, 10µM AX or 1mM L-NAME (only in B), (**D**) DAM concentration–response curve in intact or denuded pre-contracted carotid rings with and without pre-treatment with ML161, L-NAME or AX. AX: Axitinib. Colored asterisks refer to isochromatic curves and express significant differences to the maximal contraction prior to compound administration. The black asterisks (**A**–**C**) express significant differences between intact or denuded rat carotid rings. n ≥ 3. *p < 0.05, **p < 0.01, ***p < 0.001.

Intact and denuded carotid rings were used to analyze acute effects of D-amphetamine-sulfate (DAM) on vasomodulation ex vivo using six ascending DAM concentrations (0.05–1.2mM). Vasodilation of fully contracted intact carotid rings (Fig. 1) was inducible by the addition of 0.1mM DAM (5.1±1.2%, *p*=0.0011) whereas 1.2mM DAM induced maximum observed vasodilation (29.3± 2.5%, *p*<0.0001). Denuded carotid rings dilated by 10.6±2.5% after administration of 0.2mM DAM (*p*=0.0056) and 1.2mM DAM reduced constriction by 23.8±1.7% (*p*<0.0001). Intact carotid rings dilated stronger in response to DAM than the denuded carotid rings (*p*=0.0058) (Fig. 1A and Supplementary Fig. 1).

Permeability—modulating receptors and relevant molecular targets were inhibited to elucidate the mechanisms underpinning the vascular response to DAM. PAR-1, VEGF-R and NOS were blocked prior to DAM administration to identify signaling pathways in intact rat carotid rings ex vivo. No inhibitor completely blocked the DAM effect. However, after blocking PAR-1 using ML161 (Fig. 1B) a higher DAM concentration of 0.4mM (*p*=0.0368) was needed to induce vasodilation. Maximum vasodilation at 1.2mM DAM was reduced to 20.0±3.1% compared to 29.3±2.5% without blocking (*p*=0.047). Blocking VEGF-R (Fig. 1B) using Axitinib (AX) prior to DAM treatment increased initial dilation at 0.1mM DAM by 4% (*p*=0.039). N^ω^-nitro-L-arginine methyl ester (L-NAME) pre-treatment had no effect on DAM induced vasodilation in intact carotid rings (*p*>0.1). VEGF-R or PAR-1 blocking in endothelium-denuded carotid rings reduced the relaxant effect of DAM (maximum 8.7±4.7% (*p*<0.001) and 7.8±1.9% (*p*<0.001), respectively, (Fig. 1C)).

DAM concentration–response relaxation patterns differed when specific blocking was applied (Fig. 1D). Intact carotid rings without inhibitors and pre-treated with L-NAME had comparable EC_50_ values (intact: 0.275M; L-NAME: 0.295M). ML161 pre-treatment shifted the EC_50_ value to 0.338M. AX pre-treatment condensed the DAM response curve with a higher first response resulting in lower EC_50_ dose (0.201M) but with decreased maximum effect. EC_50_ values in denuded carotid rings without and with additional VEGF-R or PAR-1 blocking could not be determined due to the absent ceiling effect.

### DAM specific impedance profile in vascular cell barriers

**Figure 2:**
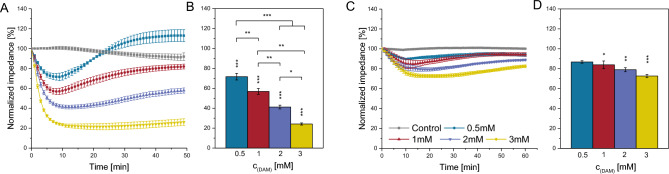
EIS *real-time* monitoring of endothelial and smooth muscle cell response following DAM addition. Normalized and averaged time traces of cellular impedance of DAM exposed HCMECs (**A**) and SMCs (**C**). Normalized maximum impedance reduction due to DAM exposure of HCMECs (**B**) and SMCs (**D**). Single asterisks indicate comparison to untreated control. Brackets indicate comparisons between experimental groups. n=3. *p<0.05, **p<0.01, ***p<0.001.

In human cardiac microvascular endothelial cells (HCMECs), electrochemical impedance spectroscopy (EIS) revealed an immediate significant, concentration—dependent decrease of the relative cellular impedance following DAM addition (Fig. 2A). Approximately eight minutes after the addition of 0.5 and 1mM DAM impedance was reduced by 28.4±3.3% (*p*<0.001) and 43.1±3.0% (*p*<0.001), respectively) (Fig. 2B). The maximum impedance reduction by 75.9±1.1% (*p*<0.001) was observed 25min after addition of 3mM DAM. DAM concentrations ≤ 1mM induced a biphasic impedance signal. In particular, DAM addition first reduced the cellular impedance and secondly enhanced the cellular impedance above control level. This effect was inversely concentration—dependent, the higher the drug concentration the weaker the biphasic impedimetric time trace. In smooth muscle cells (SMCs) DAM also decreased the relative impedance in a biphasic profile depending on the DAM concentration, whereas these effects were less pronounced than in HCMECs (*p*<0.001) (Fig. 2C and D). The maximum impedance decrease of 27.4±1.6% (*p*<0.001) in SMCs following addition of 3mM DAM corresponds to one third of the effect observed in HCMECs. Additionally, phenylephrine pre-treated SMCs showed a significantly enhanced EIS signal reduction in response to 3mM DAM (*p*=0.009; supplementary Fig. 2).

### DAM induced permeability—associated receptors and downstream effectors

**Figure 3:**
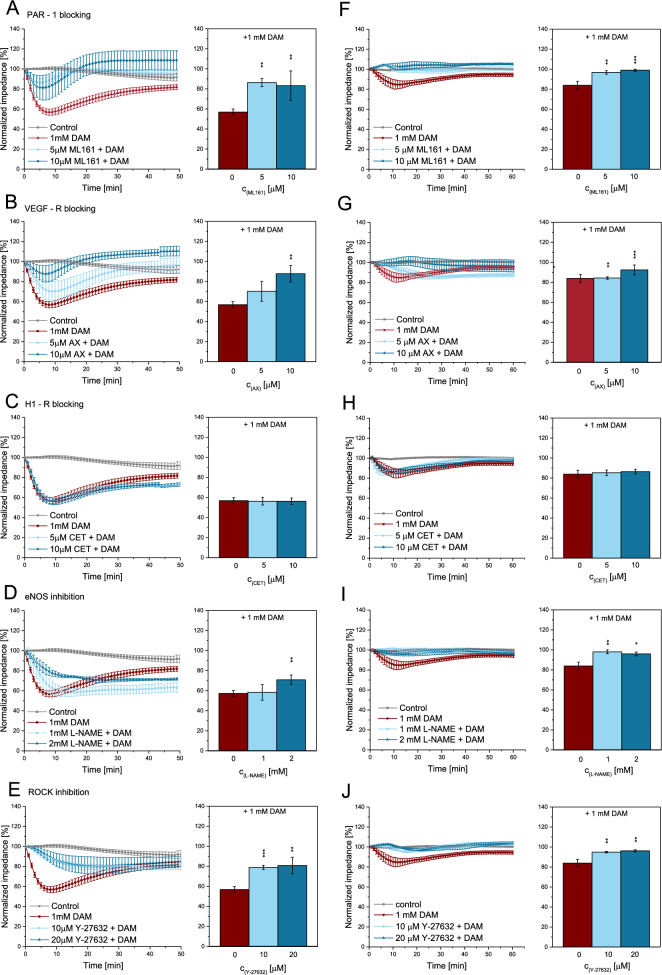
EIS *real-time* monitoring of HCMECs and SMCs following DAM addition with additional blocking of receptors and downstream targets. Normalized, averaged time traces of impedance and the maximum impedance reduction of DAM exposed HCMECs (**A**–**E**) and SMCs (**F**–**J**) with additional blocking of PAR-1 using ML161 (**A**, **F**), VEGF-R using Axitinib (AX, **G**), H1-R using cetirizine hydrochloride (CET, **C**,** H**), eNOS using L-NAME (**D**, **I**) and ROCK using Y-27632 (**E**, **J**). Due to shorter measurement duration in 1 out of 3 replicates, SEM in (**B**) is increased starting at 40 min. n=3.*p<0.05, **p<0.01, ***p<0.001.

PAR-1 blocking using ML161 in HCMECs (Fig. 3A) significantly attenuated the effect of 1mM DAM by 29% (*p*=0.0016). VEGF-R blocking in HCMECs using Axitinib (AX) attenuated the DAM induced impedance decrease by 30% (*p*=0.0036) (Fig. 3B). The biphasic impedance increase was intensified by 29% after 40min (*p*=0.0092). H1-R blocking using cetirizine hydrochloride (CET) had no effect in HCMECs and SMCs (*p*>0.9) (Fig. 3C and H). In HCMECs eNOS inhibition using L-NAME resulted in a significant suppression of the DAM response by 24% (*p*=0.001) (Fig. 3D) and cancellation of the biphasic EIS profile. ROCK blocking using Y-27632 in HCMECs diminished the effect of DAM by > 20% (*p*<0.01) (Fig. 3E). In SMCs ML161, Y-27632 or L-NAME pretreatment by trend abolished the DAM induced impedance reduction (*p*<0.01 vs. 1 mM DAM) (Fig. 3F,I,J). Blocking with 5µM AX resulted in a delayed DAM response with impeded biphasic EIS profile while 10µM AX attenuated the DAM effect completely (Fig. 3G).

### DAM and actin cytoskeleton rearrangements

**Figure 4:**
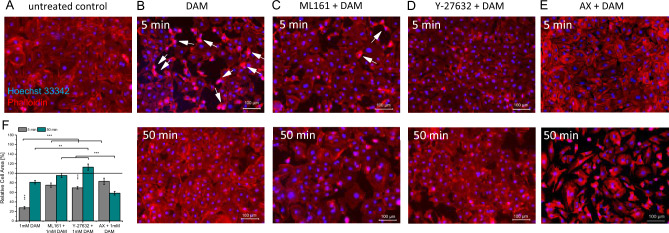
DAM mediated actin cytoskeleton rearrangement in HCMECs. F-actin and nuclei staining 5 and 50min after addition of 1mM DAM. (**A**) Untreated control, (**B**) 1mM DAM, (**C**) 10µM ML161 treatment and (**D**) 20µM Y-27632 treatment prior to DAM addition. (**E**) Quantification of the relative cell surface areas. Single asterisks indicate comparison to untreated control. Brackets indicate comparisons between experimental groups. n=3. *p<0.05, **p < 0.01, ***p < 0.001.

**Figure 5:**
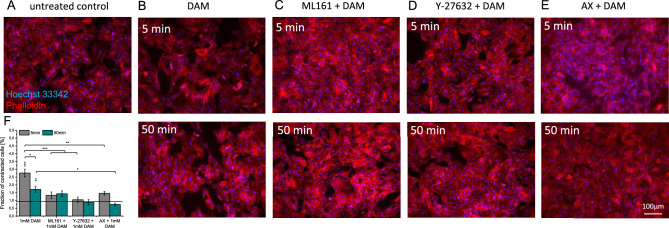
Actin cytoskeleton staining of DAM-exposed SMCs. F-actin and nuclei staining 5 and 50min after addition of 1mM DAM. (**A**) Untreated control, (**B**) 1mM DAM, (**C**) 10µM ML161 treatment, (**D**) 20µM Y-27632 and (**E**) 10µM AX treatment prior to DAM addition. (**F**) Quantification of the fraction of condensed SMCs. Single asterisks indicate comparison to untreated control. Brackets indicate comparisons between experimental groups. n=3. *p<0.05, **p<0.01, ***p<0.001.

DAM effects on HCMEC F-actin cytoskeleton were analyzed using Phalloidin staining (Fig. 4). Native HCMECs present an extensive net of actin stress fiber bundles and maintain a flat shape thereby forming a dense monolayer. A reduction in actin stress fiber bundles (Fig. 4B, upper panel) and dense Phalloidin signals close to cell nuclei were observed 5min after DAM addition. The contraction of actin fibers resulted in the breakdown of the former dense HCMECs monolayer barrier. Numerical, cell surface area showed a reduction by 71.7±3% (p<0.001) following DAM addition. The barrier was completely restored 50min after DAM addition (Fig. 4 B and F, lower panel). In ML161 or Y-27632 pre-treated HCMECs these DAM-induced changes in actin cytoskeleton were less pronounced (ML161 −24.9±4.6%, Y-27632 −30.5±2.7%; both *p*<0.001) and the flat cellular morphology was preserved (Fig. 4C,D,F). AX pre-treated HCMECs first maintained a comparably dense monolayer 5min post DAM addition (−16.9 ± 6.6%, *p*<0.001), whereas 50min post DAM the HCMEC morphology changed and the EC monolayer was disrupted (41.7±4.0%, *p*>0.001) (Fig. 4E and F). SMCs showed comparable but less pronounced effects. The fraction of contracted cells of 2.8±0.24% 5min post DAM treatment was significantly higher than in the untreated control with 0.94±0.17% contracted SMCs (*p*<0.001) (Fig. 5A,B,F). PAR-1, ROCK and VEGF-R blocking completely impeded cell morphology alterations and the formation of intercellular gaps post DAM administration (Fig. 5C–E, upper panel and F).

### eNOS phosphorylation in HCMECs

**Figure 6:**
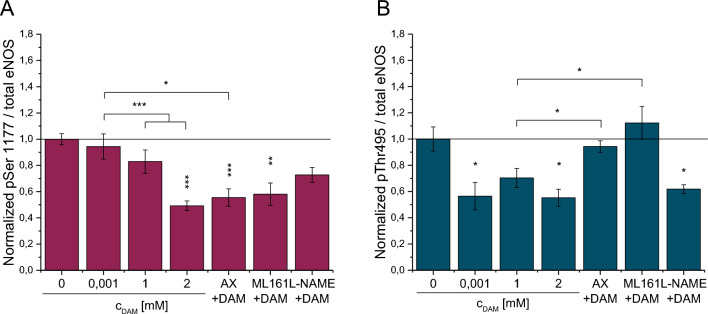
Phosphorylation at Ser1177 and Thr495 of eNOS in HCMECs in response to DAM. Ratios of phosphorylated Ser1177 (**A**) or Thr495 (**B**) 5min post DAM administration were normalized to total eNOS content in HCMECs. Untreated control was set to 100%. Single asterisks indicate comparison to untreated control. Brackets indicate the compared experimental groups. n≥3. *p<0.05, **p<0.01, ***p<0.001.

Phosphorylation patterns at eNOS Ser1177 and Thr495 in HCMECs were analyzed to conclude the enzyme activity in response to DAM (Fig. 6**, **see supplementary Figs. 3–11 for extensive Western Blot analyses). Phosphorylation of eNOS Ser1177 (pSer1177) was concentration-dependently reduced, with a maximum dephosphorylation by 50.8±3.7% 5min after administration of 2mM DAM (*p*<0.001). Dephosphorylation at Ser1177 following blocking with AX was 44.5±6.6% (*p*<0.001 vs. control) and following ML161 pre-treatment was 41.9±8.5% (*p*=0.005 vs. control), respectively. Pre-treatment with L-NAME had no effect on phosphorylation of Ser1177 (*p*=0.12).

Thr495 phosphorylation (pThr4495) was significantly decreased by 43.5±10.4% (*p*=0.033) and 44.8±6.5% (*p*=0.0103) in response to 0.001 and 2mM DAM, respectively. Pre-treatment with L-NAME prior to DAM administration did not block the DAM effect (*p*=0.85). Whereas, pre-treatment with AX or ML161 prevented DAM-induced Thr495 dephosphorylation completely (AX: *p*=0.048, ML161: *p*=0.047 vs. 1mM DAM).

## Discussion

In this study, the immediate ex vivo effects of amphetamine on arterial tone and on the major cell types of the vascular system, endothelial- and smooth muscle cells, were analyzed. In addition, the cellular signal transduction networks involved were identified. The main findings are:

1. DAM induces vasodilation in intact and denuded carotid rings ex vivo
2. DAM induces enhanced permeability in monolayers of HCMECs and SMCs
3. PAR-1 and VEGF-R are involved in DAM signaling
4. DAM induces actin cytoskeleton alterations involving ROCK signaling
5. eNOS phosphorylation is affected by DAM with potential implications for altered enzyme activity.

### Amphetamine mediated vasodilation ex vivo

In vivo*,* amphetamine and its analogues cause vasoconstriction and in general, increase peripheral resistance via norepinephrine mediated α-adrenergic stimulation^11,15^. In this study, vasodilation of intact carotid rings in response to DAM presumably via NO generation was observed. Additionally, carotid rings denuded from endothelium, merely representing the smooth muscle cell layer of the vessel, showed an acute vasodilation in response to DAM as well. Amphetamine-induced vasodilation was already described for amphetamine–based pharmaceuticals like clobenzorex^16^ in isolated aortic rings. For amphetamine-based drugs like fenproporex this effect was shown to depend on an intact endothelium^17^. Summarizing, vasodilation in acute response to amphetamines shall be considered as a general mechanism of amphetamine—based compounds.

### Amphetamine mediates enhanced endothelial permeability in vitro

Ex vivo vasodilation was further assessed in vitro using EIS where DAM induced a significant reduction of the cellular impedance signal in monolayers of HCMECs and SMCs, indicating enhanced vascular permeability^18^. In line with the ex vivo vasodilation tests, HCMECs responded significantly stronger to DAM treatment than SMCs. Accordingly, lower EC_50_ values for DAM concentration–response relation were determined in intact carotid rings and no EC_50_ values could be determined in denuded LCA rings due to an absent ceiling effect.

HCMECs and SMCs showed reversible cytoskeletal rearrangements presumably caused by actin-myosin-based cell contraction in response to DAM treatment^14^. This assumption is consistent with the prior observation that contraction of SMCs is associated with a morphological switch from round, flat cells to retracted cell bodies resulting in a reduced cellular impedance^19^. The interaction of the altered vasotonus alongside with the observed pathological cellular alterations in response to DAM remain to be verified in vivo.

### Signal transduction in amphetamine induced vasomodulation

Vasorelaxation can be induced by activation of VEGF-R as found in rat aortic preparations^20^. Disturbance of the endothelial barrier function results from reorientation of the F-actin cytoskeleton and thus results in contraction and formation of intercellular gaps. These mechanisms are triggered by Ca^2+^/Calmodulin-dependent kinase, which is activated when histamine binds to H1-R or thrombin binds to PAR-1^14^. Consequently, VEGF-R, PAR-1 and H1-R were selectively inhibited to analyze their potential involvement in DAM signaling. Inhibition of VEGF-R tyrosine kinase 1–3, using the potent and highly selective agent AX^21^ and inhibition of PAR-1 using ML161, significantly diminished effects of DAM in vitro and ex vivo*.* H1-R blocking using CET had no effects, indicating that this receptor is not involved in DAM mediated enhanced endothelial permeability. ML161 acts at the intracellular side of PAR-1 and selectively blocks G_q_^22^ mediated signal transduction via Ca^2+^/Calmodulin towards eNOS signaling^23^. ROCK inhibition blocks the signal transduction towards myosin light chain kinase phosphorylation and actomyosin contraction^24^. Blocking of PAR-1 and ROCK inhibited the DAM mediated actin cytoskeleton rearrangements and the resulting retraction of the cell bodies. Interestingly, VEGF-R was also found to activate ROCK and presumably disrupt EC focal adhesions to the actin cytoskeleton^13^. Importantly, in this study, VEGF-R blocking using AX initially enhanced cytoskeletal and functional responses to DAM but reduced the maximum effects ex vivo and in vitro*.* This observation presumably is the consequence of an accelerated and prolonged response to DAM via a complex network of VEGF-R downstream signaling cascades.

Summarizing, VEGF-R, PAR-1/G_q_ and ROCK are involved in DAM specific signaling towards actin cytoskeletal rearrangements, resulting in enhanced vascular permeability and SMC contractility^24^. Noteworthy, ROCK activation is a well-known pathomechanism in hypertension, atherosclerosis, vasospasm and stroke^24^.

PAR-1 and VEGF-R are further known to regulate eNOS activity by controlling phosphorylation status at Ser1177 and Thr495. These receptors activate protein kinase C (PKC) which then causes deactivation of eNOS by Ser1177 dephosphorylation^25^ with simultaneous preservation of basal deactivating Thr495 phosphorylation^14^. On the other hand, Akt as another downstream target of VEGF-R^26^ can counteract this process by phosphorylating Ser1177 and thus activating eNOS^27^. DAM induced a significant hypophosphorylation of both Ser1177 and Thr495, which is rather controversial since phosphorylated Ser1177 is regarded as eNOS activating and phosphorylated Thr495 is restricting eNOS activity^26^. However, recent evidence implies that phosphorylation of Ser1177 at the eNOS enzyme does not imperatively correlate with the enzymes’ activity^28^. eNOS blocking with the competitive inhibitor L-NAME, had no effect on the DAM-induced eNOS phosphorylation pattern. Importantly, VEGF-R or PAR-1 inhibition slightly diminished the DAM-induced dephosphorylation at Ser1177, but completely reversed the dephosphorylation at Thr495. These results imply that DAM specifically modulates eNOS enzyme activity by Thr495 dephosphorylation, thus promoting eNOS-driven NO generation. Although, the results imply an indirect DAM action involving PAR-1 and VEGF-R signaling, a direct interference of DAM with upstream messengers of eNOS cannot be excluded. DAM effects in endothelial cells could not be blocked completely. This is probably a consequence of the complexity of the signaling networks towards vasodilation and vascular permeability^12,29^ wherein other than the experimentally inhibited receptor signaling cascades may have compensated.

### Implications for DAM responses in the cardiovascular system

Several pathomechanisms might be underpinning the development of drug-induced heart failure. In vivo, enhanced vascular permeability is triggered by a variety of stimuli, like histamine, thrombin, VEGF or activated neutrophils^12^. As amphetamine induces enhanced vascular permeability, one severe pathological consequence may be a transendothelial extravasation of circulating cells, platelets, and serum/plasma proteins at the site of drug-induced vascular permeability. These cells may then attract and activate more platelets, which further deposit additional VEGF thus potentiating the DAM related signal transduction. This vicious cycle induced by DAM may thus result in aggregating platelets which then may quickly occlude microvascular structures^13^. Such a thrombogenic effect was reported for the contrast agent 5-Fluorouracil as a consequence of the initially direct disruption of the endothelial barrier. In addition, drug-related impaired cardiac microvasculature SMC function, namely impaired vasodilation or microvascular spasms, could result in defective adaptation in the case of changing oxygen demand and thus decreased myocardial blood supply^30^.

Importantly, we observed that the switch to a condensed cell shape resulted in the formation of intercellular gaps in the cell monolayer, which was reported to cause extravasation of fluids and macromolecules, at worst causing life-threatening edema^14^. Noteworthy, occasional case reports described pulmonary edema as side-effects of Adderall^31^ and Vyvanse, prescribed for attention-deficit hyperactivity disorder treatment^32^ and in cases of amphetamine overdosing with amphetamine serum levels up to 18mg/L^33–35^. Further, methamphetamine overdosing was reported to cause brain vascular edema and breakdown of the blood–brain-barrier in rat and mouse models^36,37^. Summarizing, these clinical observations may be underpinned by DAM induced actin cytoskeleton rearrangement and enhanced vascular permeability.

Concluding, DAM effects on vascular cells are multimodal and compensatorily transduced via PAR-1, VEGF-R involving ROCK signaling towards actin cytoskeleton breakdown and eNOS activity resulting in vasodilation and enhanced vascular permeability.

## Methods

### Drugs and inhibitors

DAM (reconstituted in Aqua dest.) was purchased from Lipomed (Arlesheim, Switzerland). L-NAME (reconstituted in Aqua dest.), ML161 (reconstituted in DMSO), Axitinib (AX, reconstituted in DMSO) and Cetirizin-hydrochloride (CET, reconstituted in Aqua dest.) were purchased from Merck (Darmstadt, Germany). Y-27632 (reconstituted in DMSO) was purchased from Miltenyi Biotech (Bergisch Gladbach, Germany).

### Ex vivo contraction force measurement of rat carotid rings

Animal procedures were performed in accordance with ARRIVE guidelines and relevant animal welfare guidelines and regulations and were approved by the local Animal Research Council, University of Leipzig and the Landesbehörde Sachsen (T 23/18). Left carotid arteries of Sprague Dawley rats (male, 11 weeks old, 450–500g, Medical-Experimental Center Leipzig, Germany) were instantly harvested after CO_2_—mediated sacrifice and transferred into carbogen (95% O_2_, 5% CO_2_)—flushed Krebs–Henseleit–Buffer (120.5mM NaCl, 4.8mM KCl, 1.2mM MgSO_4_, 1.2mM NaH_2_PO_4_, 20.4mM NaHCO_3_, 1.6mM CaCl_2_, 10mM glucose, 1mM pyruvate, pH = 7.4). Surrounding fat and connective tissue were carefully dissected and carotid vessel was cut into rings ranging from 1.5 to 2mm length. Endothelial denudation (further termed denuded) was achieved by careful insertion of a pressure catheter with a 1.4 F tip size (Millar SPR-839, ADInstruments, Sydney, Australia) prior to cutting ring segments.

Carotid rings were mounted on stainless steel pins and connected to an isometric force transducer in the DMT tissue bath system 720 MO (Hinnerup, Denmark) filled with pre-warmed Krebs–Henseleit–Buffer under continuous carbogen exposure. The buffer was changed twice followed by a 30min equilibration phase before the ring tension was manually incrementally adjusted to 20mN. Following 30min of rest the maximal contraction was acquired by adding 100mM KCl. This procedure was repeated once. Standardized experiments started with induction of an endothelium-independent contraction using 300nM phenylephrine (PE) with or without additional inhibitor addition, using 1mM L-NAME or 10µM ML161 or AX before DAM was applied incrementally (0.05–1.2mM).

### Smooth muscle cell isolation and cultivation of vascular cells

Intact rat left carotid arteries were rinsed with PBS and subjected to SMC isolation. Therefore, 2 × 2mm pieces were pressed with their luminal side onto collagen—coated tissue culture plates and incubated at 37°C, 5% CO_2_ in a tilting position for five minutes. Then, 500µL DMEM/F12 with 20% FBS and 1% penicillin/streptomycin (Thermo Fisher, Waltham, USA) were added to cover the tissue. After 4 days, medium was replaced by DMEM/F12 with 10% FBS and 1% penicillin/streptomycin. When outgrowth of cells was observed, tissue was removed and cells were expanded.

HCMECs (purchased from PromoCell, Heidelberg, Germany) of three never-smoking, male, 60–63 year-old donors were cultured in EGM_2_ (Lonza, Basel, Switzerland) supplemented with 10% FBS and 1% penicillin/streptomycin. All cells were cultured at 37°C and 5% CO_2_.

### Electrochemical impedance spectroscopic real-time monitoring

EIS was performed with an ACEA xCELLigence® Real-time Cell Analyzer (RTCA) DP instrument (ACEA Bioscience, San Diego, USA). The “E-Plates 16” are equipped with gold interdigital electrodes with approximately 80% coverage of each well bottom surface with electrodes (5mm diameter). The RTCA instrument was steadily kept in a cell culture environment. 10.000 cells per well were seeded on E-Plates and immediately applied for continuous real-time recording of impedance changes referenced to the values of the cell-free electrodes. Cells were cultivated until constant cellular impedance, indicating a confluent cellular monolayer, was reached. Cells were equilibrated in fresh medium for 24 h before compounds were added. Impedance values were recorded every 60 min prior—and every minute after compound application. To screen for compound—mediated vascular effects HCMECs and SMCs were exposed to 0.5 to 3mM DAM. ML161, AX, CET (5 and 10µM each), L-NAME (1 and 2mM) and Y-27632 (10 and 20mM) were used to block PAR-1, VEGF-R, histamine receptor 1, eNOS and ROCK, respectively. Alterations in cell indices were normalized to the cell index determined immediately before compound application.

### F-actin staining

Actin cytoskeleton alterations in HCMECs and SMCs in response to DAM with and without pre-treatment with ML161, AX or Y-26732, were analyzed using Phalloidin-iFluor 594 reagent (Abcam, Cambridge, UK). After exposure to DAM and blocking compounds for 5 or 50min, cells were fixed using 4% paraformaldehyde and F-actin fibers were stained with Phalloidin for 60min at room temperature. Nuclei were stained with 1µg/mL Hoechst 33342 (Invitrogen, Waltham, US) for 10min at room temperature. A KEYENCE BZ-X800 microscope was used for visualization. To quantify morphological changes single cell surface areas of at least 30 HCMECs per experimental condition (n=3) and fraction of contracted SMCs in relation to all SMCs in the field of view were determined using ImageJ^38^ .

### Western blot analysis

Confluent HCMECs were exposed to 0.01, 1 and 2mM DAM or PBS for 5min with and without pre-treatment with 1mM L-NAME, 10µM AX or 10µM ML161 and subsequently lysed in RIPA buffer (10mM Tris–HCl, pH 8.0, 1mM EDTA, 0.5mM EGTA, 1% Triton X-100, 0.1% sodium deoxycholate, 0.1% SDS, 140mM NaCl) containing a protease and a phosphatase inhibitor mix (Serva, Heidelberg, Germany) and sonicated. Protein concentration was determined using the BCA method (bicinchoninic acid assay, Pierce, Bonn, Germany). eNOS quantity and phosphorylation was determined using antibodies against total eNOS (Santa Cruz #sc376751, 1:200), phosphorylated Serine position 1177 (pSer1177; BD Bioscience #612393, 1:2000), and Threonine position 495 (pThr495; BD Bioscience #612706, 1:2000). Peroxidase-conjugated secondary antibodies (Sigma POD 9044 anti-mouse, 1:5000) and enzymatic chemiluminescence assay (Super Signal West Pico, Thermo Fisher Scientific Inc., Bonn, Germany) were used to visualize bands. The quantity of Ser1177- and Thr495-phosphorylated eNOS was normalized to total eNOS amount.

### Statistical analysis

Statistical analyses were performed using GraphPad Prism 6 (San Diego, CA, USA). Shapiro-Wilks-Test was performed to check for normal distribution. Data are presented as mean ± standard error of the mean (SEM). Group differences were analyzed using one-way ANOVA or student’s t-test. Time traces were assessed by two-way repeated measures ANOVA. Bonferroni correction was used for multiple testing correction. All *p*-values < 0.05 were considered statistically significant. For concentration–response curves and half-maximal effective concentration (EC_50_) calculation, the data was normalized to the maximum effect.

### Supplementary Information

Supplementary Information.

## Data Availability

The data that support the findings of this study are available from the corresponding author upon reasonable request.
