# Supplementary material for: Amphetamine increases vascular permeability by modulating endothelial actin cytoskeleton and NO synthase via PAR-1 and VEGF-R
Source: Sci Rep. 2024 Feb 13;14:3596. doi: 10.1038/s41598-024-53470-w (PMC10864289; doi:10.1038/s41598-024-53470-w)
Supplement: Supplementary file 1 — Supplementary Information. [file 41598_2024_53470_MOESM1_ESM.docx]

Amphetamine increases vascular permeability by modulating endothelial actin cytoskeleton and NO synthase via PAR-1 and VEGF-R

Supplementary Information

**Supplementary Figure 1: Quality control of LCA denudation.** Acetylcholine (ACh) induced vasorelaxation of pre-contracted intact and denuded LCA rings. Data are normalized to maximal contraction in response to 100 nM PE. vonWillebrand factor (red) and Hoechst 33342 staining (blue) of endothelial cells and nuclei. n=4. Mean ± SEM. Two-tailed student´s t-test. ***p<0.001

**Supplementary Figure 2:**  **Maximal effect of DAM exposure in smooth muscle cells.** Statistical analysis of the normalized maximal impedance reduction due to DAM exposure of SMCs with and without pre-treatment with 30 µM PE (phenylephrine). Mean ± SEM. n≥3. 2-way ANOVA with Bonferroni correction. **p<0.01.


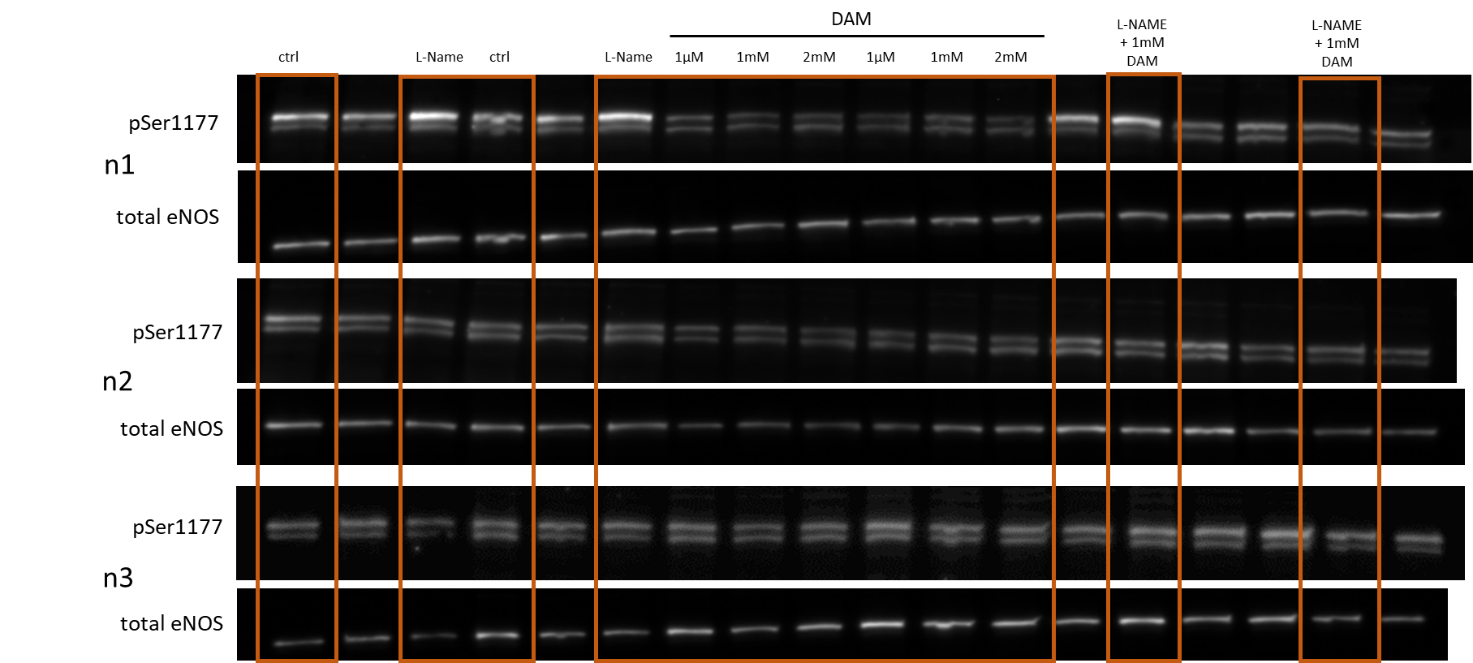


**Supplementary Figure 3: Western Blot detection of phosphorylated Ser1177 at eNOS in HCMECs.** Three independent experiments of HCMECs exposed to 1µM, 1mM and 2mM DAM for 5minutes with and without prior L-NAME treatment (1mM, 30min). Total eNOS and phosphorylated eNOS are detected at 130kDa. The bands in the orange boxes were quantified.


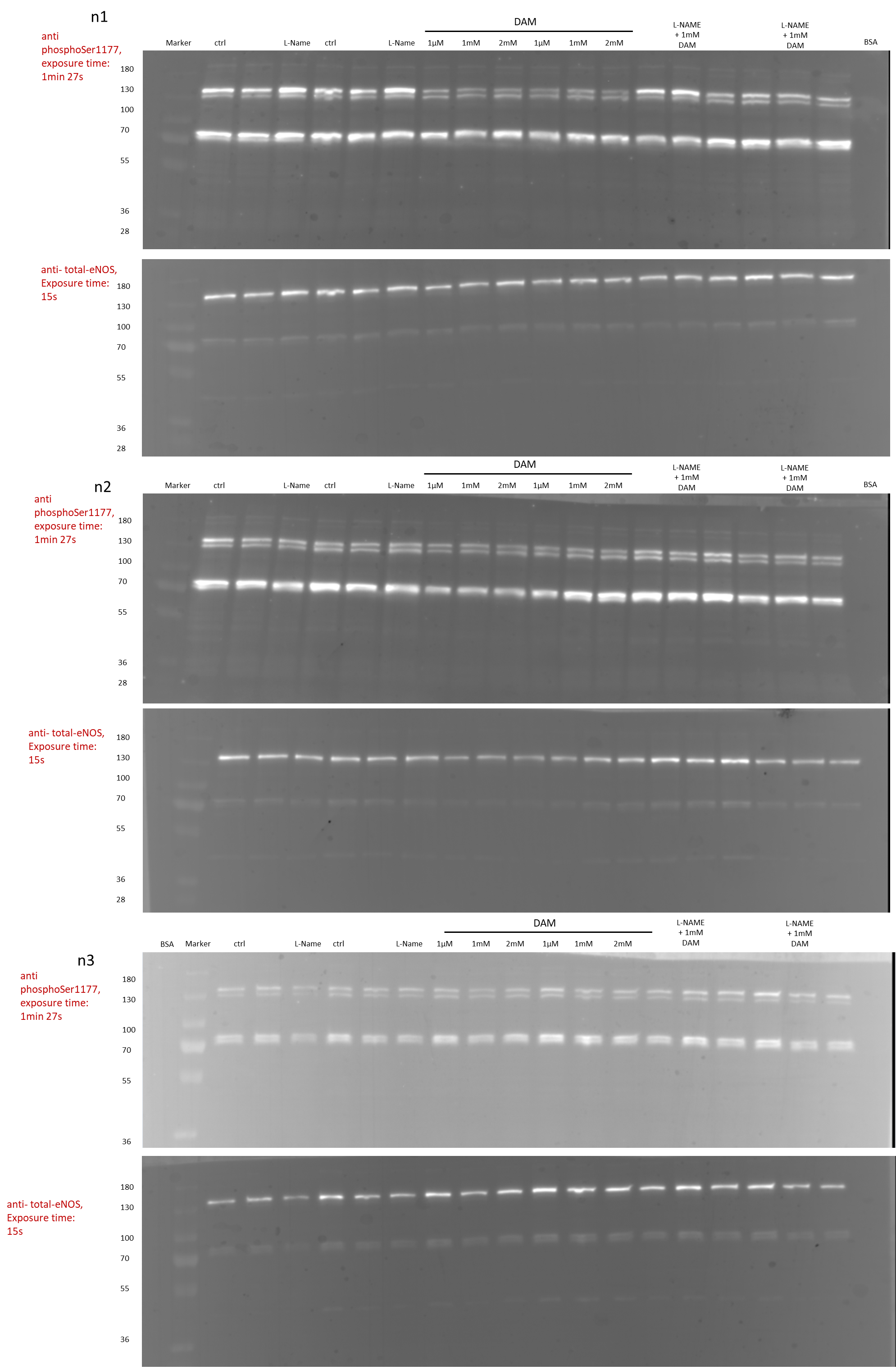


**Supplementary Figure 4:** Original Western blots of phosphorylation of Ser1177 at eNOS in DAM-treated HCMECs with and without prior L-NAME treatment. Analyzed as shown in figure 3.


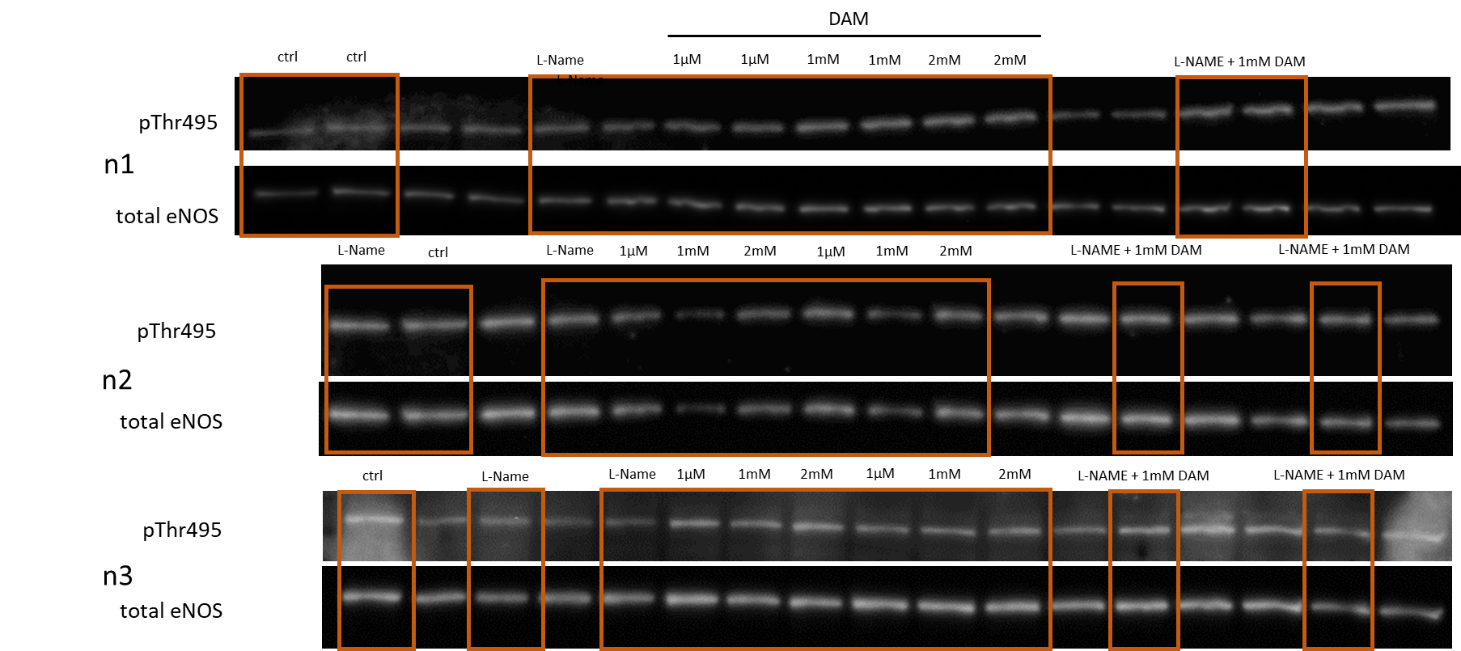


**Supplementary Figure 5:** **Western Blot detection of phosphorylated Thr495 at eNOS in HCMECs.** Three independent experiments of HCMECs exposed to 1µM, 1mM and 2mM DAM for 5minutes with and without prior L-NAME treatment (1mM, 30min). Total eNOS and phosphorylated eNOS are detected at 130kDa. The bands in the orange boxes were quantified.


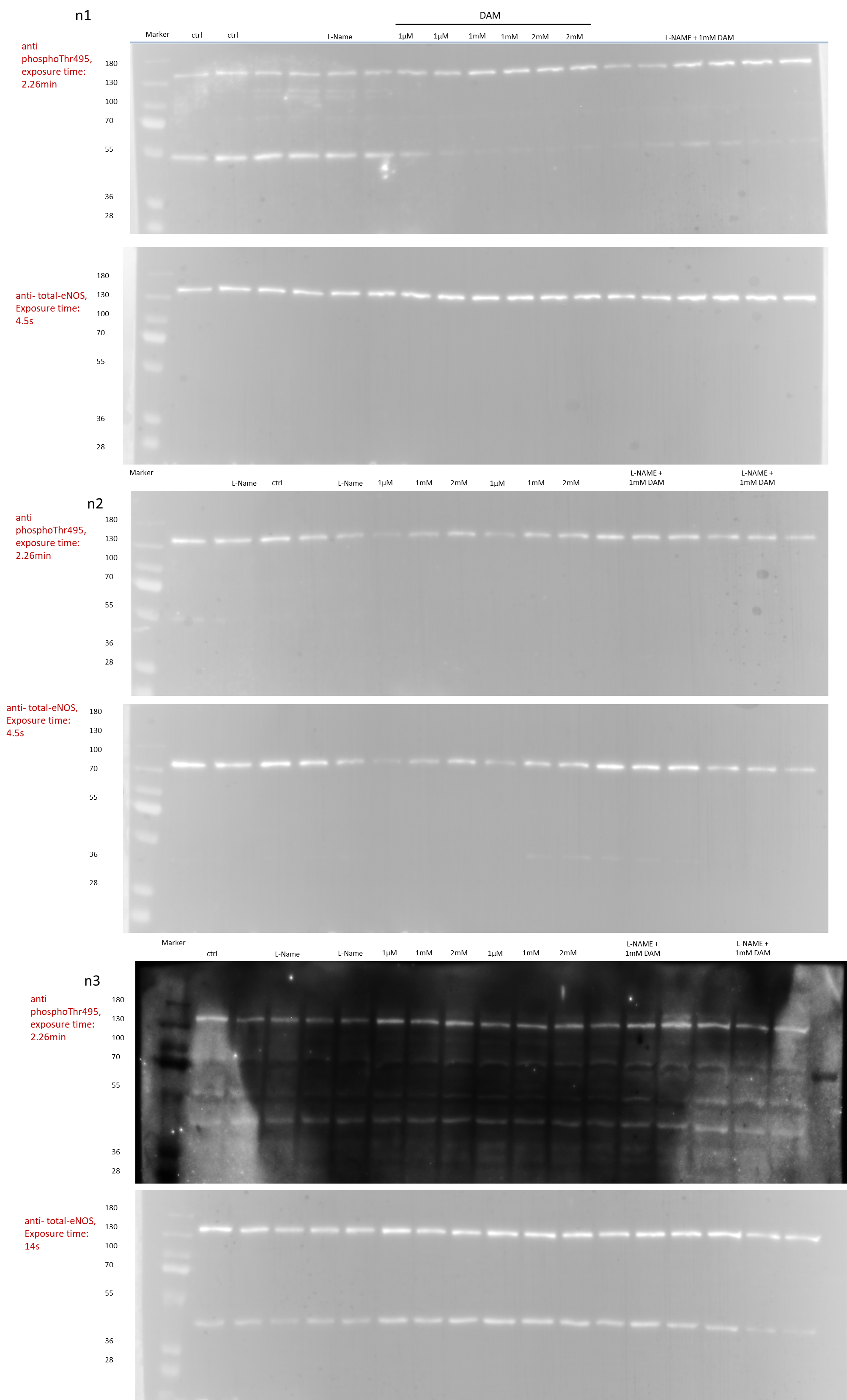


**Supplementary Figure 6:** Original Western blots of phosphorylation of Thr495 at eNOS in DAM-treated HCMECs with and without prior L-NAME treatment. Analyzed as shown in figure 5.


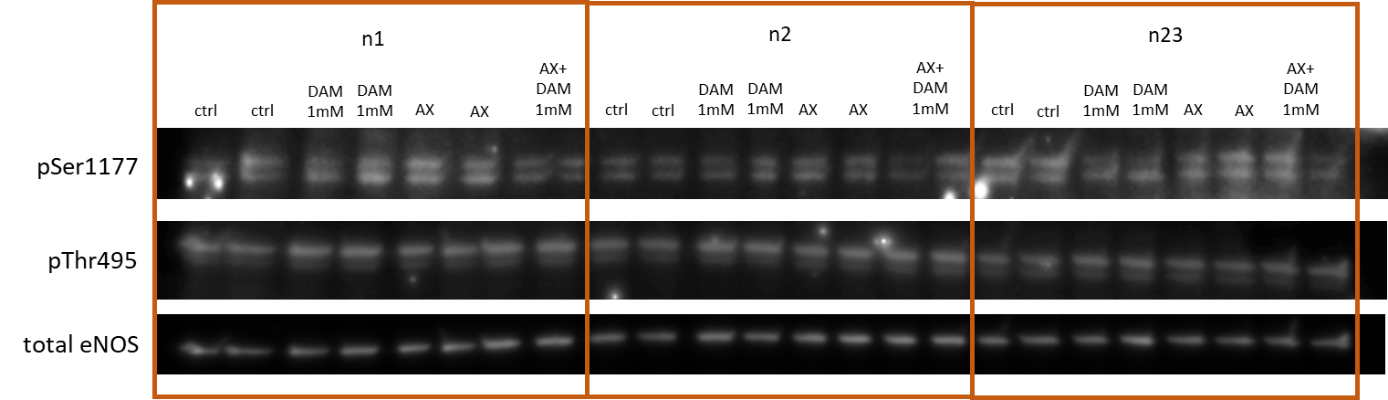


**Supplementary Figure 7: Western Blot detection of phosphorylated Thr495 and Ser1177 at eNOS in HCMECs.** Three independent experiments of HCMECs exposed to 1mM DAM for 5minutes, 10µM Axitinib (AX, 30min) and AX + 1mM DAM. Total eNOS and phosphorylated eNOS are detected at 130kDa. The bands in the orange boxes were quantified.


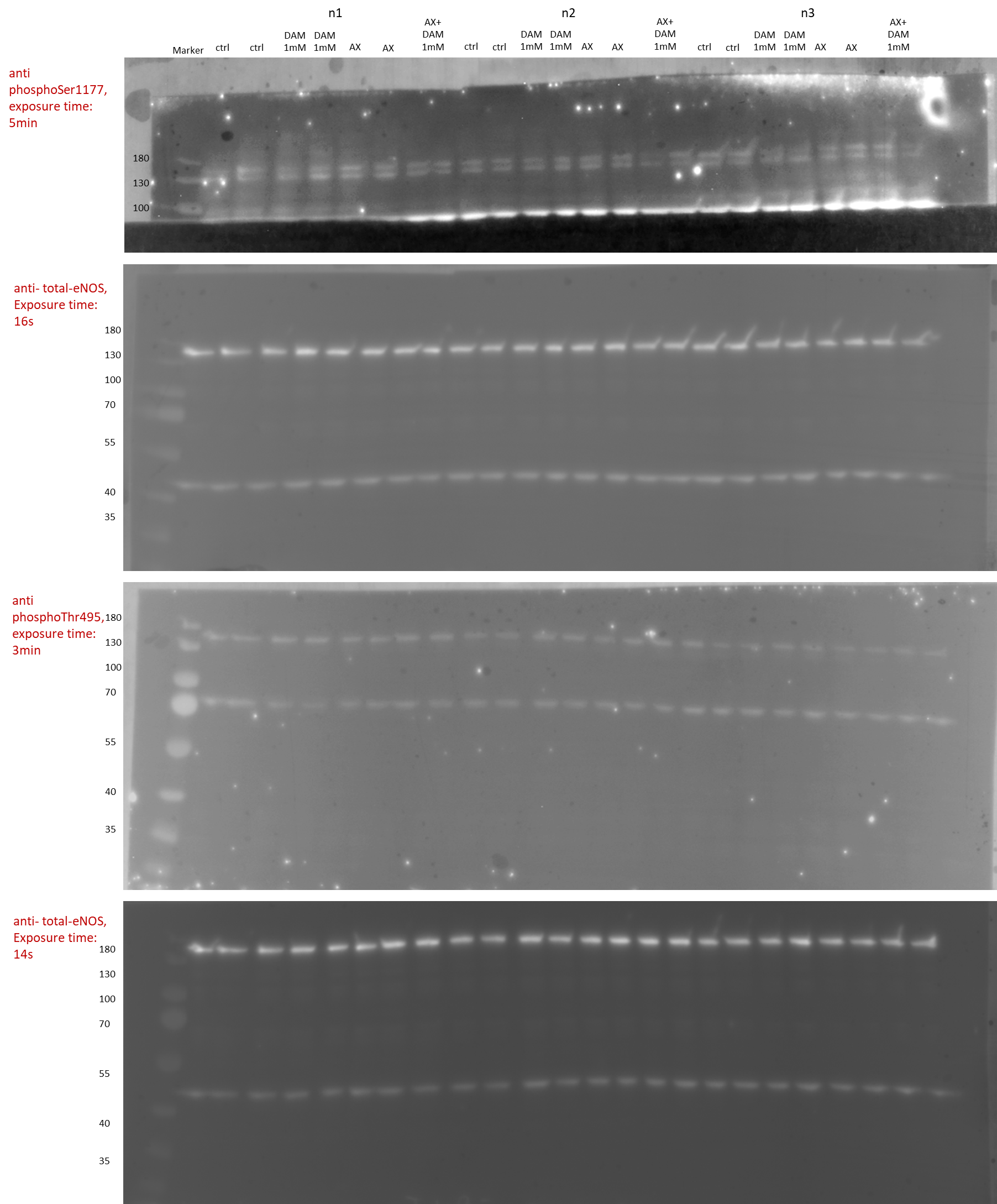


**Supplementary Figure 8:** Original Western blots of phosphorylation of Ser1177 and Thr495 at eNOS in DAM-treated HCMECs with and without prior Axitinib (AX) treatment. Analyzed as shown in figure 7. PhosphoSer1177 needed to be half - covered because of a bright band at 70kDa, which originates from BSA.


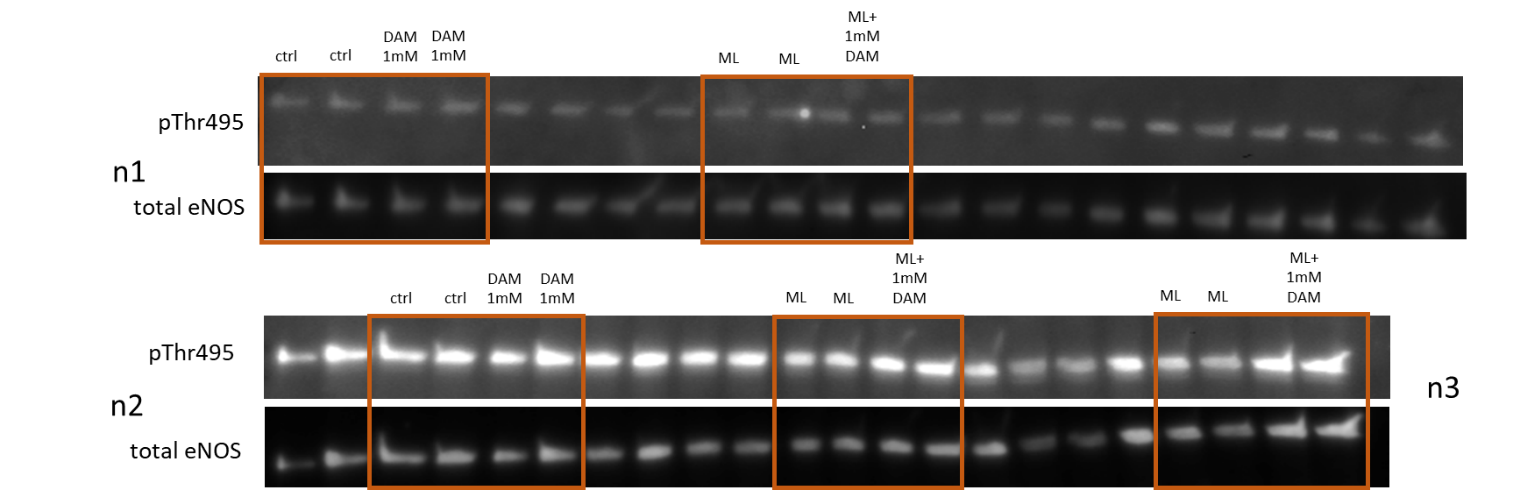


**Supplementary Figure 9: Western Blot detection of phosphorylated Thr495 at eNOS in HCMECs.** Three independent experiments of HCMECs exposed to 1mM DAM for 5 minutes, 10µM ML161 (ML, 30min) and ML + 1mM DAM. Total eNOS and phosphorylated eNOS are detected at 130kDa. The bands in the orange boxes were quantified.


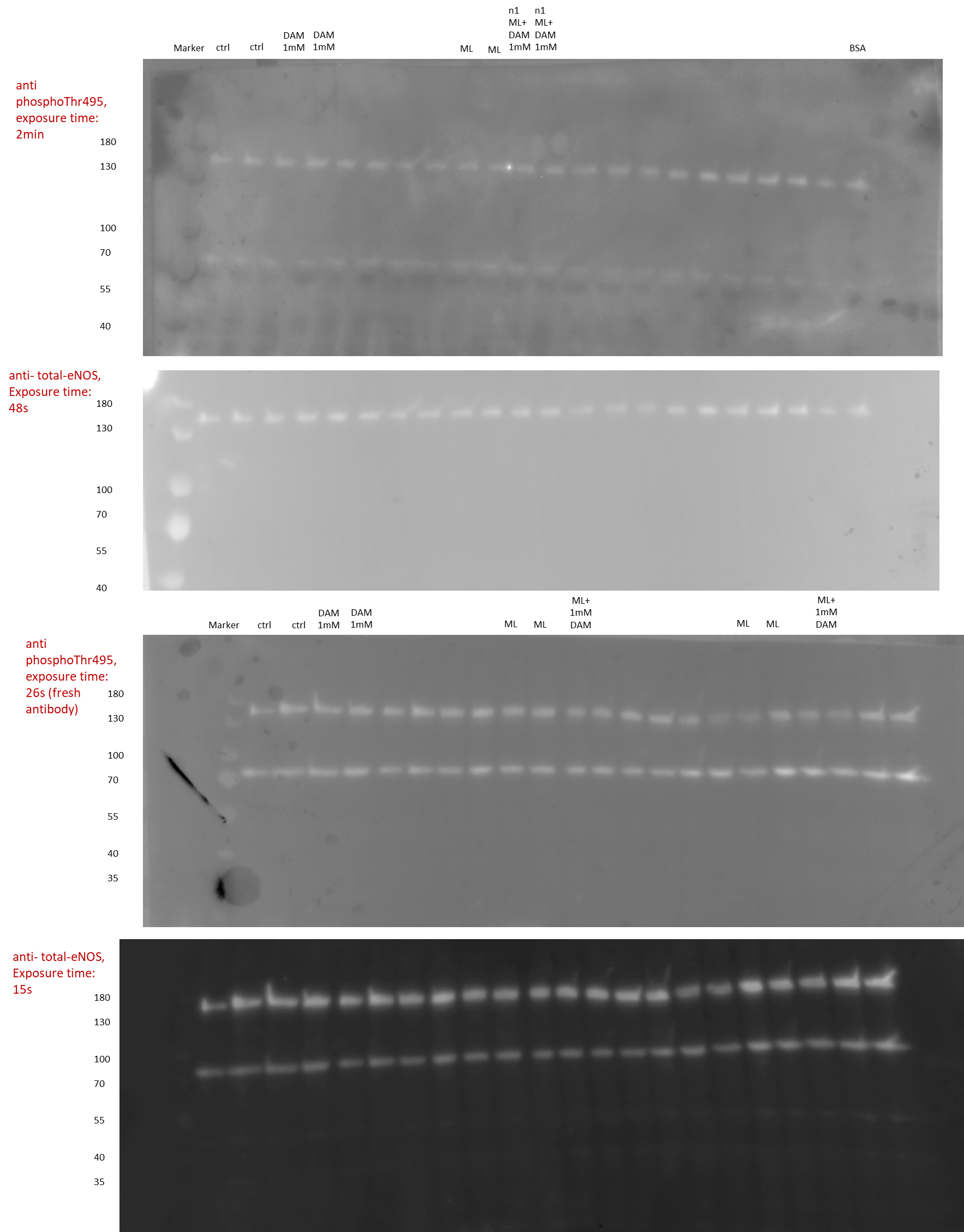


**Supplementary Figure 10:** Original Western blots of phosphorylation of Thr495 at eNOS in DAM-treated HCMECs with and without prior ML161 (ML) treatment. Analyzed as shown in figure 9.


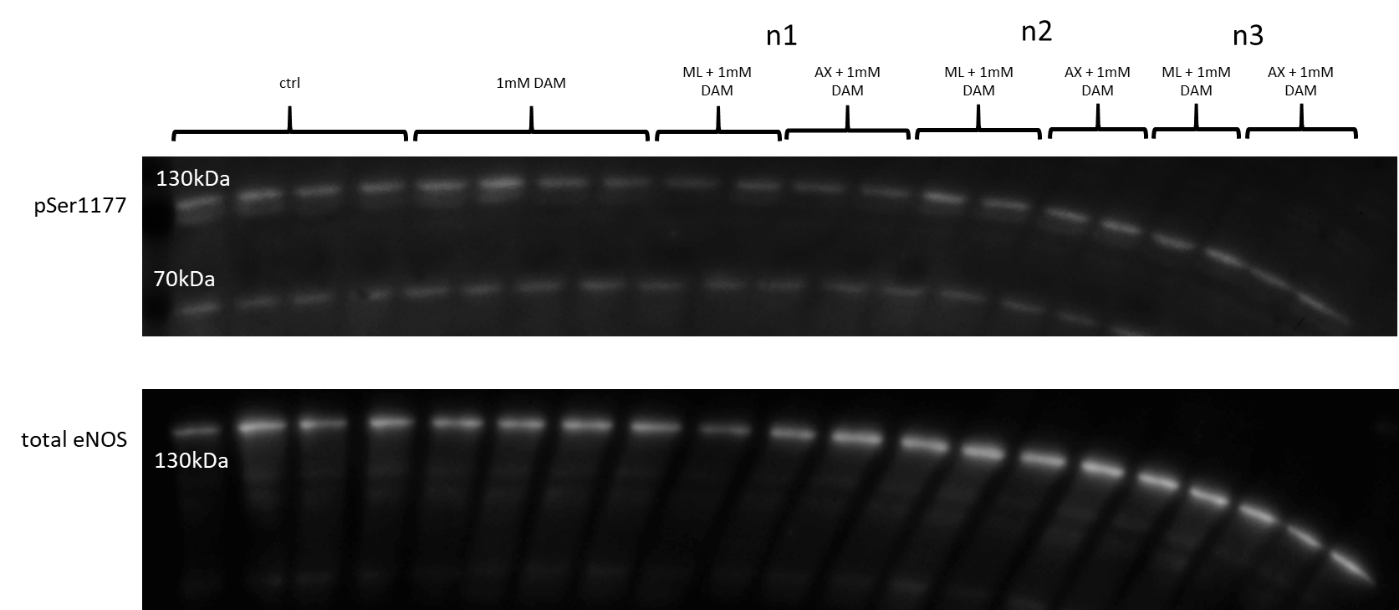


**Supplementary Figure 11:** **Western Blot detection of phosphorylated Ser1177 at eNOS in HCMECs.** Three independent experiments of HCMECs exposed to 1mM DAM for 5 minutes, 10µM ML161 (ML, 30min), 10µM Axitinib (AX, 30min) and ML + 1mM DAM or AX + 1mM DAM. Total eNOS and phosphorylated eNOS are detected at 130kDa. All bands were quantified.


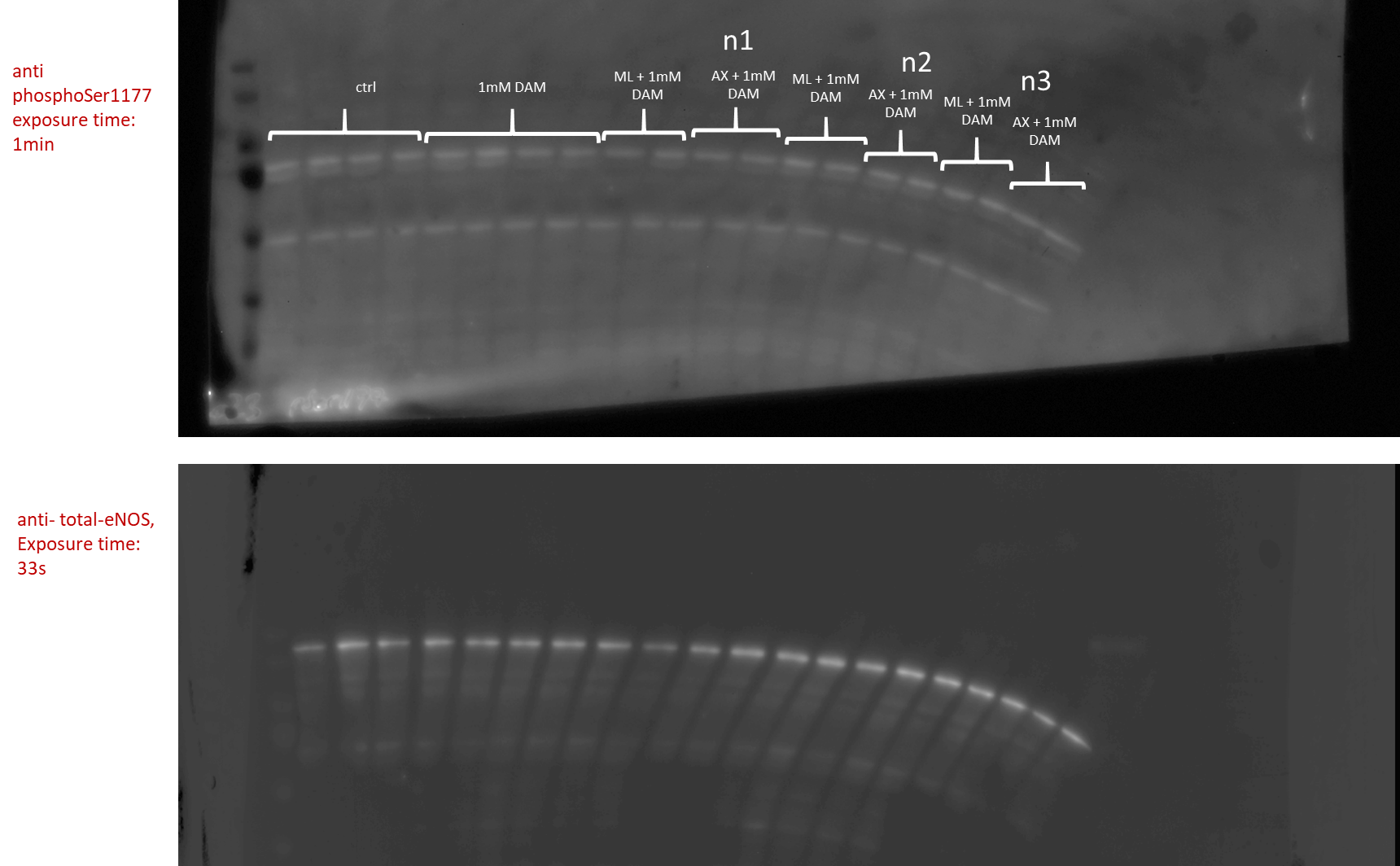


**Supplementary Figure 12**: Original Western blots of phosphorylation of Ser1177 at eNOS in DAM-treated HCMECs with and without prior ML161 (ML) treatment. Analyzed as shown in figure 11.
